# Psychiatric emergency events and risk factors among patients with severe mental disorders: an 8-year retrospective analysis in an urban district of Beijing

**DOI:** 10.3389/fpsyt.2026.1912993

**Published:** 2026-09-03

**Authors:** Yu Hu, Hongwei Wang, Mengjie Zhang, Huilin Yan, Hongfeng Yan, Qi Tian, Shurui Jin, Zhiwu Li, Zongyu Zhang

**Affiliations:** Fengtai Mental Health Center, Beijing, China

**Keywords:** community mental health services, mental disorders, physical health, retrospective studies, severe

## Abstract

**Objective:**

To investigate the characteristics and associated factors of psychiatric emergencies among patients with severe mental disorders using 8-year consecutive data from an urban district of Beijing, so as to inform evidence-based strategies for preventing and responding to such events in community mental health settings.

**Methods:**

A 1:1 age- and gender-matched case-control study was conducted. We included 349 patients who received emergency psychiatric interventions between 2018 and 2025 (case group) and 349 matched controls with no history of such events during the same period.

**Results:**

Within the case group, most participants were young-to-middle-aged males diagnosed with schizophrenia. The leading indications for intervention were risk of harm to others/public safety (36.10%) and aggressive behaviors (26.07%). Multivariable regression identified seven independent risk factors, while regular community rehabilitation was a protective factor (all *P* < 0.05). The post-COVID-19 period (2023–2025) saw a significantly higher incidence than the COVID-19 containment period (2020–2022, *P* < 0.05).

**Conclusion:**

Males, young and middle-aged adults, and schizophrenia patients are key populations for community psychiatric emergency management. These events result from combined sociodemographic, clinical, familial, and community factors. Targeted interventions can effectively reduce emergency rates, providing scientific evidence for community mental health practice.

## 1 Introduction

Mental disorders constitute the single largest contributor to the global burden of disease, representing a major public health and social crisis that inflicts profound health, economic, and social costs on individuals, families, and communities across the globe (1, 2). Patients with severe mental disorders (SMDs) typically present with behaviors that endanger the safety of themselves, others, or public property. These events are characterized by unpredictability, sudden onset, potential for violence, and severe consequences (3). Most SMDs have an insidious onset, and affected individuals frequently lack insight into their illness. Combined with pervasive family stigma and a chronic, relapsing disease course, these factors often lead to delayed recognition and intervention. As symptoms progressively worsen, patients become increasingly susceptible to engaging in various dangerous behaviors (4). As such, the prevention of high-risk behaviors and the establishment of effective emergency psychiatric response systems for individuals with SMDs have garnered growing international attention and emerged as a core component of modern community mental health practice (5).

A comprehensive understanding of the demographic and clinical characteristics of patients with SMDs who received emergency psychiatric interventions, combined with effective community-based emergency psychiatric management and the establishment of coordinated multi-sectoral long-term mechanisms, is critical for reducing the incidence of dangerous behaviors among this population (6).

This study analyzed demographic data, clinical characteristics, treatment histories, and emergency intervention details of patients with SMDs who received emergency psychiatric interventions in an urban district of Beijing between 2018 and 2025. The primary objective was to examine the patterns and associated factors of psychiatric emergencies in this population, to inform evidence-based strategies for preventing psychiatric crises and improving community mental health services.

## 2 Methods

### 2.1 Study population

We conducted a retrospective 1:1 individually matched case-control study. The case group comprised all 349 eligible patients with SMDs who received formal emergency psychiatric interventions in an urban district of Beijing between 2018 and 2025.

The control candidate consisted of all registered SMD patients who received routine community follow-up in the same district throughout the study period (2018–2025). All candidates were recorded in the Beijing Municipal Mental Health Management System and had no documented history of formal psychiatric emergency interventions. All such events are formally registered and flagged in the Emergency Management Module of the Beijing Municipal Mental Health Management System. Patients with any flagged record of formal psychiatric emergency intervention were automatically excluded from the control candidate pool by the system.To ensure consistent baseline eligibility and maximum comparability between groups, the control group adhered to the same inclusion and exclusion criteria as the case group, with the sole exception of no prior psychiatric emergency intervention records.

Each case entry was individually matched to one control on a 1:1 basis according to three pre-specified criteria: identical residential community, same gender, and age within ±3 years at the index date. Notably, patients who experienced multiple psychiatric emergency episodes were repeatedly enrolled based on each independent emergency event, with a unique matched control randomly selected according to the patient’s age, gender, and residential community at the time of each emergency occurrence. Eligible controls were randomly selected without replacement from the community candidate pool. A total of 349 matched case-control pairs were ultimately included in the analysis.

Emergency psychiatric intervention was defined as a free medical service provided by a multidisciplinary team comprising psychiatric clinicians, police officers, and community workers to patients with SMDs who had an identified need for emergency care or exhibited a risk of violent or disruptive behavior.

All study data were retrieved from the Beijing Municipal Mental Health Management System. Collected variables covered general demographic characteristics (gender, age, marital status, educational level, and employment status), disease duration, family history of mental illness, family caregiving status, and social functioning. Additional clinical and follow-up indicators were also extracted, including disability level, participation in community rehabilitation services, number of prior psychiatric hospitalizations, and receipt of free psychiatric treatment. Notably, for patients in the case group, all follow-up data were sourced from the most recent daily follow-up record prior to their psychiatric emergency event; for controls, follow-up data were obtained from their last daily follow-up record in 2025 to ensure consistent and baseline assessment across the study population.

Furthermore, under the national Specifications for the Management and Treatment of Patients with Severe Mental Disorders (2018 Edition), patients in the case group received identical routine follow-up frequency as community-managed patients (potential controls) before the occurrence of psychiatric emergency events. Additionally, we explicitly excluded potential control candidates with prior histories of violent behavior, alcohol or substance misuse, persecutory delusions, threats toward others, expressed homicidal ideation, antisocial conduct, marked emotional lability, or ongoing severe life stressors. Therefore, controls in our study did not receive more intensive follow-up services compared with cases prior to their emergency events, and follow-up frequency was comparable across both groups.

### 2.2 Ethical approval

This retrospective observational study was conducted using legally acquired, fully de-identified administrative data from the Beijing Municipal Mental Health Management System. No direct contact with study participants occurred, and no interventions were performed that could affect their clinical care or daily lives.

All personally identifiable information (including names, national identification numbers and contact details) was permanently removed prior to analysis. Only de-identified clinical and follow-up records were retained, and strict data confidentiality protocols were implemented in accordance with medical ethics principles. Designated study staff were responsible for data processing; all datasets were encrypted and securely stored, and only de-identified data relevant to the study were used for statistical analysis.

The study was conducted in strict accordance with the ethical principles of the Declaration of Helsinki, two regulatory guidelines:as well as two domestic regulatory requirements: the Guidelines for Ethical Review of Biomedical Research Involving Human Subjects in Beijing Medical and Health Institutions (Jing Wei Ke Jiao (2018) No. 24) and the Measures for Ethical Review of Life Science and Medical Research Involving Human Subjects (Guo Wei Ke Jiao Fa (2023) No. 4).

The study protocol was formally reviewed by the Ethics Committee of Beijing Fengtai District Mental Health Center (Beijing Fengtai District Psychiatric Prevention and Treatment Hospital), which formally granted a waiver of full ethical review for the study, with the official approval number Lunshen (2026) No. 4. The Ethics Committee is formally registered with the Beijing Fengtai District Health Commission (Institutional Registration No.: EC-20231206-1003) and fully complies with China’s national ethical review regulations for medical research involving human subjects. The institution does not conduct pharmaceutical clinical trials, and this non-interventional retrospective observational study falls fully within the scope of its ethical review authority.

Given that this is a retrospective observational study using de-identified routine medical record data, with no additional interventions or extra risks imposed on participants, the requirement for written informed consent was also formally waived by the Ethics Committee.

### 2.3 Inclusion criteria

1. Patients who received formal emergency psychiatric interventions met either of the following two criteria:

Diagnosed with one of the severe mental disorders specified in the International Classification of Diseases, 10th Revision (ICD-10), including schizophrenia, schizoaffective disorder, paranoid psychosis, bipolar disorder, mental disorder due to epilepsy, and mental retardation with concomitant mental disorders;

Were identified under Article 30, Paragraph 2, Subparagraph 2 of the Mental Health Law of the People’s Republic of China, and clinically diagnosed and assessed as having SMDs, not limited to the six aforementioned diagnoses.

Suspected cases were eligible if they presented with one or more symptoms listed in the checklist for identifying abnormal mental and behavioral symptoms from the 2018 Edition of the National Standards for the Management and Treatment of Patients with Severe Mental Disorders.

2. Age ≥ 18 years;

3. Registered and under regular follow-up management at community health service centers.

### 2.4 Exclusion criteria

1. Were unable to complete the required study follow-up assessments;
2. Refused to receive regular community mental health management;
3. Had other conditions that made them ineligible for participation in this study.
4. Had more than 20% of key data missing from their formal emergency psychiatric intervention records, including basic demographic information, intervention indications, and intervention process details.

### 2.5 Statistical analysis

This study employed a 1:1 matched case-control study design, with matching variables including residential community, gender, and age (within 3 years). All statistical analysis were performed using IBM SPSS Statistics version 26.0.

Categorical variables with two categories were compared using McNemar’s test for paired data. Unordered categorical variables with more than two categories were compared using Bowker’s test of symmetry.

Ordered categorical variables were compared using the Wilcoxon signed-rank test. Continuous variables that followed a normal distribution (assessed by the Shapiro-Wilk test) were presented as mean ± standard deviation (SD), and between-group comparisons were performed using the paired t-test.The goodness-of-fit of the regression model was evaluated using the Hosmer-Lemeshow test. Multicollinearity between independent variables was assessed using the variance inflation factor (VIF).

Variables with a P value< 0.05 in univariate analysis were entered into a multivariate conditional logistic regression model to identify independent risk factors for formal emergency psychiatric interventions in patients with SMDs. All statistical tests were two-tailed, with a significance level set at α = 0.05. A P value < 0.05 was considered statistically significant.

## 3 Results

### 3.1 Characteristics of emergency psychiatric interventions

Among the 349 patients with SMDs who received formal emergency psychiatric interventions, the most common indication was risk of harm to public safety or others, which occurred in 126 (36.10%) cases. This was followed by actual harm to public safety or others (91, 26.07%) and disease relapse with clinical deterioration (101, 28.94%). Together, these three indications accounted for more than 90% of all interventions. Acute or severe adverse drug reactions (16, 4.58%) and self-harm or suicidal behavior/risk (15, 4.30%) were less common indications.

Regarding intervention measures, emergency psychiatric hospitalization was the most frequently used approach, implemented in 184 (52.72%) cases. On-site temporary intervention was the second most common measure, used in 161 (46.13%) cases. Only 4 (1.15%) cases received other measures, including police detention. Detailed information is presented in **Table 1**.

**Table 1:** Indications and measures of formal emergency psychiatric interventions.

Item	Number of cases (n=349)	Percentage (%)
Indications for intervention		
Self-harm or suicidal behavior/risk	15	4.30
Acute or severe adverse drug reactions	16	4.58
Disease relapse with clinical deterioration	101	28.94
Risk of harm to public safety or others	126	36.10
Actual harm to public safety or others	91	26.07
Intervention measures		
On-site temporary intervention	161	46.13
Emergency psychiatric hospitalization	184	52.72
Other measures– Police detention	4	1.15

### 3.2 Annual distribution of formal emergency psychiatric intervention cases

The temporal distribution of formal emergency psychiatric intervention events from 2018 to 2025 is presented in **Table 2**.

**Table 2:** Temporal distribution of formal emergency psychiatric intervention events [n (%)].

Quarter (Month range)	2018	2019	2020	2021	2022	2023	2024	2025	Total
Q1 (Jan-Mar)	20(45.45)	5(29.41)	13(29.55)	8(40.00)	4(10.81)	16(35.56)	8(12.31)	30(38.96)	104(29.80)
Q2 (Apr-Jun)	15(34.09)	5(29.41)	19(43.18)	6(30.00)	4(10.81)	12(26.67)	15(23.08)	22(28.57)	98(28.08)
Q3 (Jul-Sep)	6(13.64)	4(23.53)	8(18.18)	4(20.00)	16(43.24)	14(31.11)	25(38.46)	17(22.08)	94(26.93)
Q4 (Oct-Dec)	3(6.82)	3(17.65)	4(9.09)	2(10.00)	13(35.14)	3(6.67)	17(26.15)	8(10.39)	53(15.19)
Total	44	17	44	20	37	45	65	77	349

A Pearson chi-square goodness-of-fit test was applied to compare the seasonal distribution of recorded events. The test was conducted under the null hypothesis of equal expected event frequencies across the four seasons and results reflect event distribution patterns rather than population-level incidence differences. The results showed a statistically significant difference in event proportion among different seasons (χ^2^=18.51, *P* < 0.001), with significantly higher proportion rates in spring and summer than in winter.

Specifically, proportion of total events in spring (29.80%) and summer (28.08%) was significantly higher than that in winter (15.19%) (both adjusted P < 0.05). No statistically significant differences were observed between spring and summer, or between autumn and winter (both adjusted P > 0.05). Detailed information is shown in **Table 3**.

**Table 3:** Seasonal distribution of formal emergency psychiatric intervention events .

Quarter (Season)	Observed number of events (n)	Proportion (%)	Chi-square contribution (χ^2^)
Q1 (Spring)	104	29.80***	3.21
Q2 (Summer)	98	28.08**	1.32
Q3 (Autumn)	94	26.93	0.52
Q4 (Winter)	53	15.19	13.46
Total	349	100	18.51

A Pearson chi-square goodness-of-fit test was performed under the null hypothesis of equal expected event frequencies across seasons (χ²=18.51, df=3, P<0.001). All expected frequencies were >5, meeting the application conditions of the chi-square test.*Post-hoc* pairwise comparisons with winter as the reference group were adjusted using the Bonferroni method (3 comparisons in total, adjusted significance threshold α′ ≈ 0.017): **P<0.01, ***P<0.001. All proportions represent the share of total recorded events, not population incidence rates.

Overall, the number of formal emergency psychiatric intervention events among patients with SMDs showed a fluctuating upward trend from 2018 to 2025, with two distinct abnormal low points: 2019 (17 cases) and 2021 (20 cases).

The relatively low number of events in 2019 was primarily attributed to the comprehensive upgrade and transition of the Beijing Municipal Mental Health Management Information System, which resulted in partial data loss and delayed reporting during the system handover period.

Although the missing data are concentrated in the baseline period and do not affect the core comparative analysis between the 2020–2022 containment phase and the 2023–2025 post-pandemic phase, they may introduce a certain degree of bias in the estimation of the overall 6-year time trend. Therefore, findings involving the 2019 baseline should be interpreted with caution.

In 2021, Beijing implemented strict COVID-19-related community-wide lockdown and home quarantine measures, which significantly reduced outdoor activities and exposure to external stimuli among patients with SMDs. Meanwhile, community health service centers strengthened home delivery of medications and remote follow-up services for homebound patients, enabling timely intervention for some cases of clinical deterioration and thus reducing the occurrence of emergency psychiatric intervention events in the short term.

**Figure 1:**
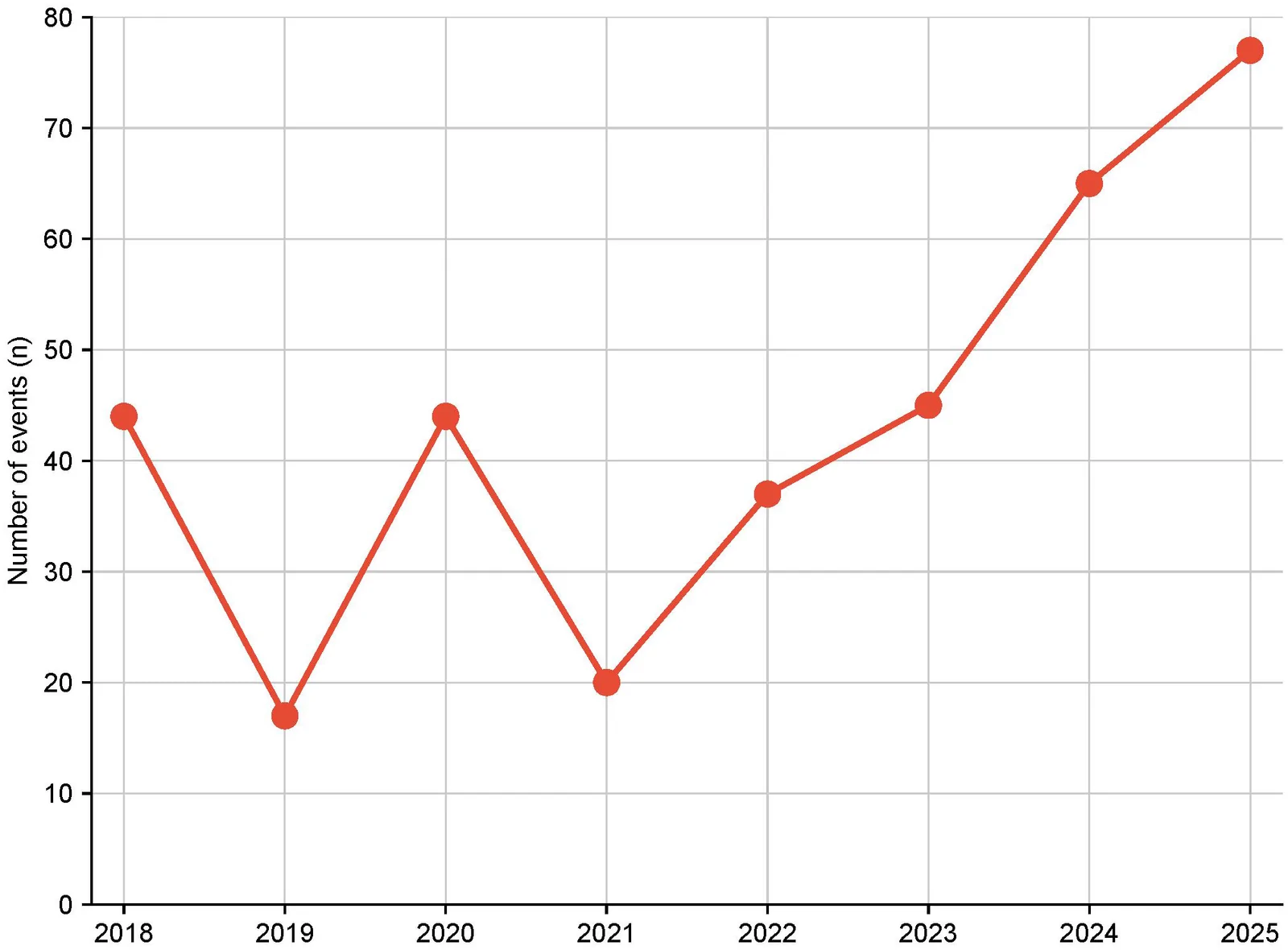
Annual trend of formal emergency psychiatric intervention events.

After excluding these two abnormal data points, the overall trend continued to show a fluctuating upward pattern in recorded event counts, as illustrated in **Figure 1**.

### 3.3 Risk assessment of patients during emergency psychiatric interventions

Among the 349 patients with SMDs who received formal emergency psychiatric interventions, risk assessment results showed that Grade 1 risk behavior was the most common, occurring in 150 (42.98%) cases. This was followed by Grade 0 in 64 (18.34%) cases, Grade 2 in 28 (8.02%) cases, Grade 3 in 30 (8.60%) cases, Grade 4 in 39 (11.17%) cases, and Grade 5 in 38 (10.89%) cases. Detailed results are presented in **Table 4**.

**Table 4:** Patient risk assessment during emergency interventions.

Risk grade	Description of risk behavior	Number of cases [n (%)]
Grade 0	None of the behaviors listed in Grades 1–5	64 (18.34)
Grade 1	Verbal threats or shouting without any destructive behavior	150 (42.98)
Grade 2	Destructive behavior limited to the home, targeting property, and can be persuaded to stop	28 (8.02)
Grade 3	Obvious destructive behavior targeting property, occurring in any setting, and cannot be persuaded to stop	30 (8.60)
Grade 4	Persistent destructive behavior targeting property or people, occurring in any setting, and cannot be persuaded to stop	39 (11.17)
Grade 5	Any violent behavior against people using controlled dangerous weapons, or acts such as arson and explosion	38 (10.89)

### 3.4 Analysis of factors associated with emergency psychiatric interventions and sociodemographic characteristics

This study enrolled a total of 349 patients with SMDs who received formal emergency psychiatric interventions (case group). Using a 1:1 matched case-control study design, 349 patients with SMDs who had not experienced any formal emergency psychiatric interventions were selected as the control group, matched on residential community, gender, and age (within 3 years).

There were no statistically significant differences in the distribution of matching factors (age, gender) and non-matching factors (education level, disease duration) between the two groups (all *P* > 0.05), indicating good comparability.

Paired statistical analysis revealed statistically significant differences between the two groups in disease diagnosis, marital status, economic status, employment status, number of previous hospitalizations, disability type, family guardianship capacity, and participation in community rehabilitation services (all *P* < 0.05).

Specifically, the case group had significantly higher proportions of patients with schizophrenia, poverty rate, unemployment rate, number of previous hospitalizations, rate of mental disability, and proportion of inadequate family guardianship capacity than the control group, while the participation rate in community rehabilitation services was significantly lower. Detailed results are presented in **Table 5**.

**Table 5:** Comparison of baseline clinical and sociodemographic characteristics.

Characteristic	Case group (n=349)	Control group (n=350)	P value
Age [n (%)]			0.803
≤35 years	51 (14.61)	45 (12.89)	
36–59 years	219 (62.75)	224 (64.18)	
≥60 years	79 (22.64)	80(22.92)	
Gender [n (%)]			0.939
Male	196 (56.16)	195 (55.87)	
Female	153 (43.84)	154 (44.13)	
Diagnosis [n (%)]			<0.001
Schizophrenia	233 (66.76)	176 (50.43)	
Bipolar disorder	76 (21.78)	89 (25.50)	
Mental disorder due to epilepsy	5 (1.43)	21 (6.02)	
Schizoaffective disorder	3 (0.86)	3 (0.86)	
Intellectual disability with mental disorders	16 (4.58)	56 (16.05)	
Other psychotic disorders	16 (4.58)	4 (1.15)	
Marital status [n (%)]			<0.001
Unmarried	163 (46.70)	156 (44.70)	
Married	109 (31.23)	158 (45.27)	
Widowed	15 (4.30)	4 (1.15)	
Divorced	62 (17.77)	31 (8.88)	
Economic status [n (%)]			0.006
Impoverished	55 (15.76)	31 (8.88)	
Non-impoverished	294 (84.24)	318 (91.12)	
Education level [n (%)]			0.197
High school or below	243 (69.63)	227 (65.04)	
College or above	106 (30.37)	122 (34.96)	
Employment status [n (%)]			<0.001
Unemployed	215 (61.60)	193 (55.30)	
Retired	100 (28.65)	80 (22.92)	
Employed	34 (9.74)	76 (21.78)	
Number of previous hospitalizations [n (%)]			<0.001
0	61 (17.48)	187 (53.58)	
1	63 (18.05)	64 (18.34)	
2–3	109 (31.23)	62 (17.77)	
≥4	116 (33.24)	36 (10.32)	
Disability status [n (%)]			<0.001
Mental disability	253 (72.49)	176 (50.43)	
Intellectual disability	14 (4.01)	47 (13.47)	
Unassessed	82 (23.50)	126 (36.10)	
Family guardianship capacity [n (%)]			<0.001
Adequate	259 (74.21)	319 (91.40)	
Partially adequate	39(11.17)	17 (4.87)	
Inadequate	51 (14.61)	13 (3.72)	
Disease duration [n (%)]			0.107
<10 years	288 (82.52)	271 (77.65)	
≥10 years	61 (17.48)	78 (22.35)	
Participation in community rehabilitation services [n (%)]			0.013
Yes	241 (69.05)	270 (77.36)	
No	108 (30.95)	79 (22.64)	

### 3.5 Analysis of factors associated with emergency psychiatric intervention events

Variable coding criteria for the multivariable conditional logistic regression analysis in this study are presented below, which define the coding rules and reference groups for the dependent variable and all included independent variables. All variables were numerically coded to meet the requirements of regression analysis.

The dependent variable was formal emergency psychiatric intervention status, which was binary coded as 0 for the control group (no emergency intervention occurred) and 1 for the case group (emergency intervention occurred).

All independent variables were categorical variables. Coding methods and reference groups were determined based on variable characteristics and study logic, as detailed in **Table 6**.

**Table 6:** Variable coding scheme for multivariable conditional logistic regression analysis.

Variable description	Coding scheme
Formal emergency psychiatric intervention status	0 = Control group, 1 = Case group
Diagnosis	0 = Schizophrenia (reference), 1 = Bipolar disorder, 2 = Mental disorder due to epilepsy, 3 = Intellectual disability with mental disorders, 4 = Schizoaffective disorder, 5 = Other psychotic disorders
Marital status	0 = Married (reference), 1 = Unmarried, 2 = Widowed, 3 = Divorced
Economic status	0 = Non-impoverished (reference), 1 = Impoverished
Employment status	0 = Employed (reference), 1 = Unemployed, 2 = Retired
Number of previous hospitalizations	0 = 0 (reference), 1 = 1, 2 = 2–3, 3 = ≥4
Disability status	0 = Unassessed (reference), 1 = Mental disability, 2 = Intellectual disability
Family guardianship capacity	0 = Adequate (reference), 1 = Partially adequate, 2 = Inadequate
Participation in community rehabilitation services	0 =Did not participate (reference), 1 = Participated

For categorical variables with small cell counts (including schizoaffective disorder under the diagnosis category and widowed status under the marital status category), all original clinical categories were retained in the primary model for full exploratory presentation. Sensitivity analysis were further performed by merging sparse categories into clinically similar subgroups to verify the robustness of the results, and no material changes in the significance or direction of core variables were observed after merging.

### 3.6 Multivariable analysis of factors associated with emergency psychiatric interventions

A total of 349 matched case-control pairs were included in the final multivariable conditional logistic regression model. Multivariable conditional logistic regression analysis revealed that being widowed, being divorced, impoverished status, unemployment, increased number of previous hospitalizations, mental disability, and inadequate family guardianship capacity were statistically significant independent risk factors for formal emergency psychiatric intervention events in patients with SMDs. In contrast, participation in community rehabilitation services was a statistically significant independent protective factor, as detailed in **Table 7**.

**Table 7:** Multivariable analysis of emergency interventions.

Variable	Subgroup	Reference group	*β*	SE	OR (95% CI)	*P* value
Diagnosis	Bipolar disorder	Schizophrenia	−0.315	0.181	0.73 (0.51~1.04)	0.075
	Mental disorder due to epilepsy	Schizophrenia	−1.238	0.372	0.29 (0.14~0.60)	0.001
	Schizoaffective disorder	Schizophrenia	−0.105	0.578	0.90 (0.29~2.81)	0.842
	Intellectual disability with mental disorders	Schizophrenia	−1.561	0.279	0.21 (0.12~0.36)	<0.001
	Other psychotic disorders	Schizophrenia	1.019	0.452	2.77 (1.14~6.72)	0.024
Marital status	Unmarried	Married	0.247	0.169	1.28 (0.92~1.78)	0.141
	Widowed	Married	0.888	0.423	2.43 (1.07~5.53)	0.034
	Divorced	Married	0.708	0.213	2.03 (1.34~3.08)	0.001
Economic status	Impoverished	Non-impoverished	0.482	0.201	1.62 (1.09~2.40)	0.016
Employment status	Unemployed	Employed	0.631	0.221	1.88 (1.22~2.90)	0.004
	Retired	Employed	0.392	0.239	1.48 (0.92~2.37)	0.104
Number of previous hospitalizations	1	0	0.559	0.2	1.75 (1.18~2.59)	0.005
	2–3	0	1.008	0.202	2.74 (1.85~4.06)	<0.001
	≥4	0	1.381	0.22	3.98 (2.58~6.13)	<0.001
Disability status	Mental disability	Unassessed	0.718	0.18	2.05 (1.44~2.92)	<0.001
	Intellectual disability	Unassessed	−1.022	0.293	0.36 (0.20~0.64)	<0.001
Family guardianship capacity	Partially adequate	Adequate	0.678	0.252	1.97 (1.20~3.23)	0.007
	Inadequate	Adequate	0.952	0.271	2.59 (1.52~4.41)	<0.001
Participation in community rehabilitation services	Participated	Did not participate	−0.416	0.165	0.66 (0.48~0.91)	0.015

Collinearity diagnostics were conducted for all independent variables entered into the model. The maximum variance inflation factor (VIF) was 1.87, which was below the conventional threshold of 5, indicating no significant multicollinearity among the included variables.

Specifically, compared with patients with schizophrenia, patients with mental disorder due to epilepsy and intellectual disability with mental disorders had a significantly lower risk of emergency events, while patients with other psychotic diagnoses had a 2.77-fold higher risk. Although there was a trend toward reduced risk in patients with bipolar disorder, the difference was not statistically significant.

In terms of sociodemographic characteristics, compared with married patients, widowed and divorced patients had a significantly increased risk of emergency events. Impoverished patients had a 1.62-fold higher risk than non-impoverished patients, and unemployed patients had a 1.88-fold higher risk than employed patients.

Regarding disease characteristics, the number of previous hospitalizations was significantly positively correlated with the risk of emergency events: compared with patients with no previous hospitalizations, the risk increased sequentially in patients with 1, 2–3, and ≥4 hospitalizations.

With respect to family and community factors, compared with patients with adequate family guardianship capacity, those with partially adequate and inadequate guardianship capacity had a significantly increased risk. Compared with patients who did not participate in community rehabilitation services, those who participated had a 34% lower risk of emergency events.

### 3.7 Pre- vs. Post-COVID-19 subgroup analysis of emergency psychiatric events

Given the anomalous data points in the overall time trend, an exploratory subgroup analysis was conducted to describe temporal changes in emergency psychiatric events in patients with SMDs across different phases of the COVID-19 pandemic. The 6-year study period was split into two equal 3-year epochs: the strict containment era (2020–2022) and the post-pandemic era (2023–2025).

The results showed that a total of 187 emergency intervention events occurred during 2023–2025, which was significantly higher than the 101 events during 2020–2022. The average annual number of events increased from 33.7 to 62.3, representing an increase of 84.9%.

There were statistically significant differences in the reasons for intervention between the two phases. Specifically, the proportion of emergency intervention events due to self-harm or suicidal behavior/risk was significantly higher during the COVID-19 containment period, while the proportion of events due to disease relapse and deterioration of mental status was significantly higher during 2023–2025.

## 4 Discussion

Formal emergency psychiatric interventions for patients with severe mental disorders (SMDs) are an essential component of community mental health services. Their occurrence is closely associated with multiple factors including patients’ clinical characteristics, social support, and disease management, and directly impacts patient safety, family well-being, and social stability.

This study conducted a systematic analysis of the clinical data, temporal distribution, risk assessment results, and influencing factors of 349 patients with SMDs who received formal emergency psychiatric interventions, with 349 patients who did not experience emergency events serving as controls. We identified the occurrence characteristics, independent risk factors, and protective factors of emergency interventions, providing scientific evidence for optimizing emergency management strategies for SMDs and reducing the incidence of emergency events.

The present study showed that among patients receiving formal emergency psychiatric interventions, those with schizophrenia were the most common, predominantly young and middle-aged adults (36–59 years), and males outnumbered females. These findings are consistent with previous studies showing that violent and disruptive events in patients with SMDs are more common in males and young and middle-aged adults, and that patients with schizophrenia are more likely to exhibit dangerous behaviors (7–9). These findings help define the core high-risk populations for targeted community mental health management.

In terms of the reasons and measures for formal emergency psychiatric interventions, the main triggers in patients with SMDs were behaviors or risks endangering public safety or the safety of others, followed by disease relapse and deterioration of mental status. Emergency inpatient treatment in specialized psychiatric hospitals was the primary intervention measure. These findings are consistent with previous studies. Most patients were classified as Grade 1 in risk assessment, which underscores the clinical value of early identification and intervention for patients with low-grade risky behaviors in community settings (10).

Analysis of temporal distribution characteristics showed that the 349 formal emergency psychiatric intervention events peaked in spring and summer. Combined with clinical practice and relevant studies, the main reasons are speculated as follows: First, the hot and humid weather in spring and summer easily induces negative emotions such as irritability and anger in patients, leading to emotional outbursts and thus increasing the probability of emergency events (11). Second, the large difference in day and night duration in summer leads to reduced average sleep time and food intake in patients, which may cause disease relapse in some patients with SMDs and subsequent behaviors endangering public safety or the safety of others (12). Third, the seasonal transition in spring is accompanied by significant changes in temperature and air pressure, which easily affect patients’ physical and mental status, trigger disease relapse, and ultimately lead to formal emergency psychiatric intervention events (13). This seasonal pattern provides an evidence base for time-stratified risk prevention in routine community management.

In terms of sociodemographic characteristics, the results of the multivariable conditional logistic regression analysis in this study showed that being widowed, being divorced, poverty, and unemployment were independent risk factors for emergency events in patients with SMDs. Patients with unstable marital status lack spousal emotional support and daily supervision, making it difficult to detect and intervene in disease fluctuations in a timely manner. Impoverished and unemployed patients not only face greater economic pressure but also have weaker social support networks and are more vulnerable to social stigma. These factors collectively increase their risk of dangerous behaviors (14). These findings highlight the necessity of targeted social security and assistance for socioeconomically vulnerable patients with SMDs.

Regarding variable selection for the multivariable model, covariates were determined based on clinical relevance and prior empirical evidence rather than solely on univariate statistical significance. The included variables cover three core domains: sociodemographic characteristics, clinical disease features, and family-community support factors, which ensures that clinically important confounders are retained in the model. We acknowledge the potential overlap among diagnosis, disability status, number of previous hospitalizations, and participation in community rehabilitation services, as more severe disease conditions may be associated with higher disability levels, more frequent hospitalizations, and higher priority for community rehabilitation services. Collinearity diagnostics showed that the maximum variance inflation factor (VIF) was 1.87, indicating that multicollinearity was within the acceptable range. To further verify result robustness, we performed a sensitivity analysis using a clinically selected parsimonious covariate set, and the direction and significance of core risk and protective factors remained largely unchanged, supporting the reliability of the main findings.

This suggests that government departments should further strengthen treatment and assistance efforts for patients with SMDs, improve the medical security and social assistance systems, and focus on the living and medical needs of patients with unstable marital status, poverty, and unemployment.

Notably, following the full adjustment of COVID-19 pandemic prevention and control policies, the incidence rate of formal emergency psychiatric intervention events in patients with SMDs increased significantly compared with the pandemic containment period, and presented characteristics distinct from those during the pandemic.

The pandemic containment period was mainly characterized by an elevated risk of self-harm and suicide, which was associated with stringent social isolation, reduced access to medical services, and heightened psychological stress among patients during this period (15).

In contrast, psychiatric emergency events in the post-pandemic era were predominantly associated withdriven by disease relapse and worsening psychiatric status, which may suggest a potential lagged impact of the COVID-19 pandemic on patients with SMDs. However, this temporal comparison is exploratory in nature and cannot establish a causal relationship between pandemic policy transitions and the increase in events, as the aggregated event counts may also be affected by changes in reporting practices, service availability, population size, and healthcare-seeking behavior. Cumulative gaps in routine treatment and impaired social functioning during the pandemic, combined with mounting socioeconomic pressure in the post-pandemic phase, may have contributed to the concentrated surge in emergency events (16, 17).

In addition, this study found that patients with inadequate family guardianship capacity were at higher risk of emergency events in the post-pandemic era. This finding suggests that following the conclusion of public health emergencies, greater priority should be given to the medical and social support needs of vulnerable groups with weak guardianship, and that long-term follow-up and intervention mechanisms should be established to prevent disease relapse and high-risk behaviors.

Notably, as this study adopts a retrospective observational case-control design, the identified associations are statistical in nature and cannot be interpreted as definitive causal relationships. Accordingly, policy implications derived from the findings should be applied with appropriate caution. our results also support the value of alternative health system investments, including strengthened early identification of disease relapse, improved continuity of routine psychiatric care, assertive community follow-up, and targeted interventions to promote treatment adherence. These approaches all carry important clinical and public health significance for reducing psychiatric emergency events.

We prioritize the four policy strategies outlined below for two key reasons. First, they align directly with the independent risk and protective factors identified in our multivariate regression analysis, with the strongest empirical support from the present dataset. Second, they are highly compatible with China’s existing community mental health management framework and can be implemented with strong operational feasibility.

Building on these findings, four priority policy strategies are proposed. First, stratified and precise management of key high-risk populations should be strengthened to enable proactive intervention. Second, the family guardianship support system should be improved, as insufficient guardianship capacity was confirmed as a prominent risk factor, especially in the post-pandemic period. Third, the coverage of community rehabilitation services should be expanded, given that regular community rehabilitation was identified as an independent protective factor against emergency events. Fourth, a cross-sectoral mental health emergency response mechanism for public health emergencies should be established to mitigate the lagged health impact of major public health events. In parallel to these core strategies, sustained investment in early relapse detection, continuous care delivery, assertive community follow-up, and treatment adherence promotion is also recommended to build a comprehensive multi-level prevention and control system. Collectively, these measures can effectively reduce the incidence of psychiatric emergency events and safeguard the safety of both patients and the general public.

## 5 Limitations

This study has several limitations that should be acknowledged. First, this was a retrospective observational study conducted in a single urban district of Beijing, and the matched case-control design can only identify statistical associations rather than definitive causal relationships. Second, all data were derived from administrative records of the community mental health management system, which carry potential risks of information misclassification and incomplete record-keeping. In particular, partial data loss occurred during the upgrade and transition of the municipal mental health information system in 2019, which may introduce bias to the estimation of temporal trends in the baseline period. Third, the study lacked detailed information on medication adherence, substance use, objective symptom severity, physical comorbidities, and specific levels of family and social support, which may lead to residual confounding. Fourth, over the 8-year study period, changes in emergency service availability, reporting thresholds and healthcare-seeking behavior may have influenced the number of recorded events, making it difficult to fully separate real changes in incidence from changes in reporting practices. Finally, all subjects were recruited from a single district, and the findings may have limited generalizability to other regions, rural areas, or different healthcare systems.

In conclusion, the occurrence of formal psychiatric emergency interventions among patients with SMDs stems from the complex interaction of sociodemographic, clinical, familial, and community-level factors. Key high-risk populations identified in this study include patients with schizophrenia, those with multiple prior psychiatric hospitalizations, patients with mental disability, those with inadequate family guardianship capacity, as well as individuals with unstable marital status, economic hardship, and unemployment.

Future research may adopt prospective cohort designs to verify the causal relationships between the identified factors and psychiatric emergency events, and to evaluate the real-world effectiveness of targeted intervention strategies in community settings.

## Data Availability

The datasets presented in this study can be found in online repositories. The names of the repository/repositories and accession number(s) can be found in the article/supplementary material.
